# Supplementary material for: Is There a FADS2-Modulated Link between Long-Chain Polyunsaturated Fatty Acids in Plasma Phospholipids and Polyphenol Intake in Adult Subjects Who Are Overweight?
Source: Nutrients. 2021 Jan 21;13(2):296. doi: 10.3390/nu13020296 (PMC7909565; doi:10.3390/nu13020296)
Supplement: Supplementary file 1 [file nutrients-13-00296-s001.pdf]

Supplementary Materials

Figure S1. Selection of study subjects and study flow.

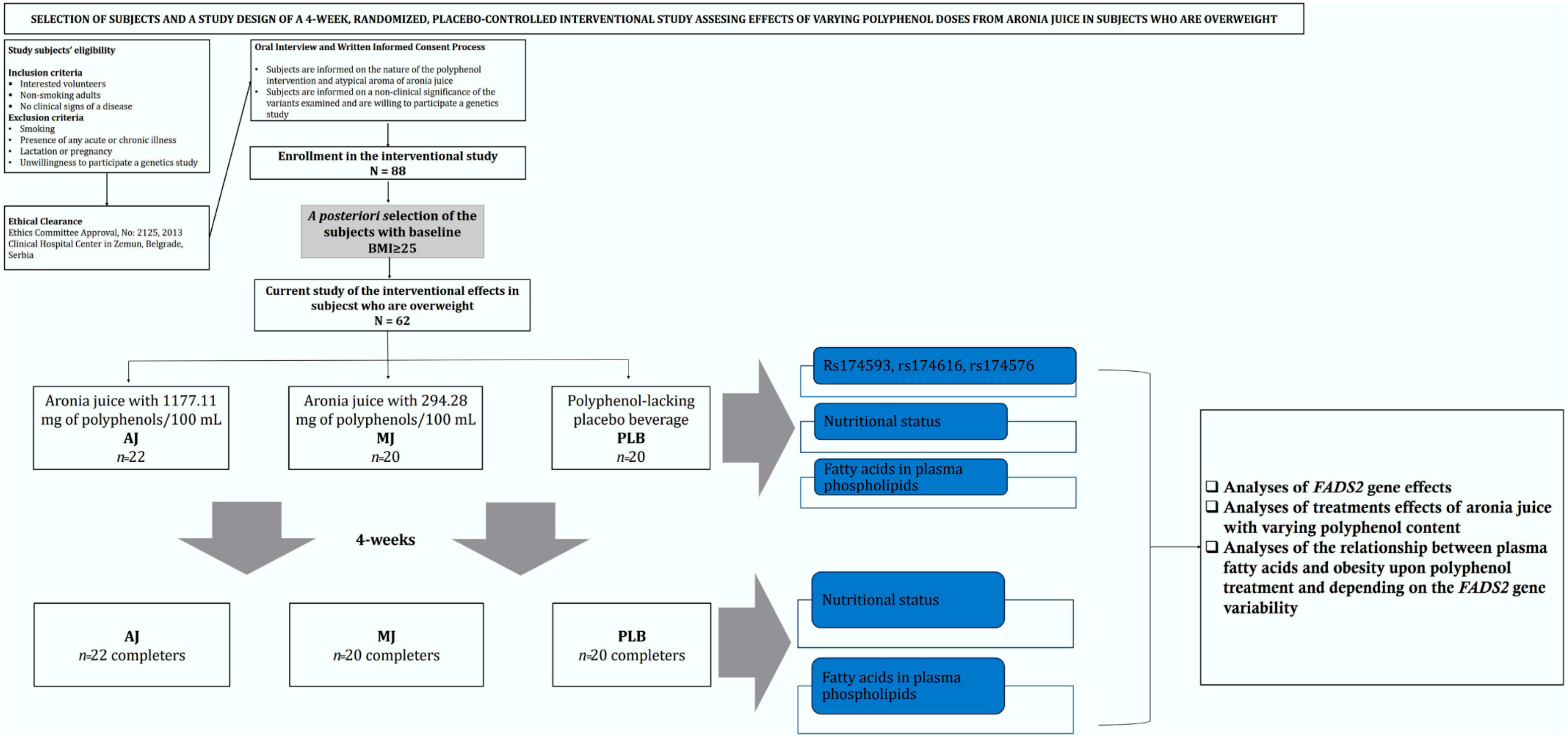

Table S1. Baseline daily dietary intake of study subjects who are overweight (BMI≥25) across the study intervention groups with varying polyphenol intake.

	Total	AJ	MJ	PLB
	n= 62	*n*=22	*n*=20	*n*=20
Carbohydrates, g	221.34±8.75	222.79±12.45	204.95±12.74	236.07±19.84
Protein, g	80.82±2.84	82.29±4.64	80.09±4.28	79.87±5.99
Fat, g	83.60 [68.54, 113.59]	82.61 [70.16, 113.94]	83.02 [72.71, 100.75]	94.98 [57.40, 120.09]
SFA, g	28.75±1.44	29.41±2.63	29.76±2.31	26.98±2.57
MUFA, g	24.89 [22.10, 35.44]	25.37 [21.76, 36.06]	24.45 [22.81, 32.62]	23.18 [19.20, 44.63]
PUFA, g	22.67 [16.47, 31.15]	21.08 [15.95, 31.29]	24.55 [19.47, 28.90]	26.12 [14.45, 38.68]
C12:0, g	0.84 [0.51, 1.19]	1.04 [0.50, 1.55]	0.88 [0.49, 1.10]	0.67 [0.51, 1.09]
C14:0, g	2.04 [1.44, 3.03]	2.29 [1.42, 3.07]	2.29 [1.49, 3.59]	1.72 [1.24, 2.82]
C16:0, g	14.40±0.70	14.78±1.21	14.60±1.14	13.74±1.32
C18:0, g	6.65 [4.61, 8.58]	6.31 [4.50, 9.05]	6.57 [5.48, 8.17]	7.14 [3.22, 9.71]
C18:1n-9, g	20.74 [17.03, 27.26]	21.11 [17.34, 27.72]	20.12 [17.19, 24.02]	19.41 [14.69, 34.72]
C18:2n-6, g	17.96 [14.85, 23.98]	17.10 [13.60, 24.61]	19.64 [17.19, 22.97]	16.49 [12.62, 26.52]
C18:3n-3, g	0.70 [0.50, 1.24]	0.66 [0.50, 1.24]	0.73 [0.49, 1.34]	0.76 [0.47, 1.28]
C20:4n-6, g	0.15 [0.10, 0.20]	0.17 [0.11, 0.23]	0.14 [0.09, 0.20]	0.12 [0.10, 0.21]
C20:5n-3, g	0.04 [0.01, 0.14]	0.07 [0.02, 0.23]	0.04 [0.01, 0.24]	0.03 [0.01, 0.10]
C22:6n-3, g	0.18 [0.10, 0.43]	0.20 [0.09, 0.39]	0.14 [0.10, 0.43]	0.18 [0.08, 0.46]
Total polyphenols, mg	952.05±44.73	959.66±64.42	966.70±87.88	928.58±85.63

Data are presented as Mean ± SEM or Median [25th, 75th percentile] depending on a distribution. Variance in distribution of the parameters was evaluated by the general linear model and Kruskal-Wallis analyses, for parametric and non-parametric approach, respectively; testing differences between parameter distributions across the study treatments with varying polyphenol content ($p>0.05$ for all parameters).

AJ, subjects drinking aronia juice with 1177.11 mg of polyphenols per 100 mL; MJ, subjects drinking aronia juice with 294.28 mg of polyphenols per 100 mL; PLB, subjects drinking polyphenol-lacking placebo beverage. SFA, Total saturated fatty acid; MUFA, Total monounsaturated fatty acid; PUFA, Total polyunsaturated fatty acid; C12:0, Lauric acid; C14:0, Myristic acid; C16:0, Palmitic acid; C18:0, Stearic acid; C18:1 n-9, Oleic acid; C18:2 n-6, Linoleic acid; C18:3 n-3, α-Linolenic acid; C20:4 n-6, Arachidonic acid; C20:5 n-3, Eicosapentaenoic acid; C22:6 n-3, Docosahexaenoic acid.

Table S2. Baseline levels of individual fatty acids in plasma phospholipids of subjects who are overweight (BMI≥25) across the study intervention groups with varying polyphenol intake.

	**Total** *n*= 62	**AJ** *n*=22	**MJ** *n*=20	**PLB** *n*=20
Palmitic, C16:0	30.39±0.28	30.08±0.58	30.17±0.40	31.02±0.38
Palmitoleic, C16:1n-7	0.59±0.03	0.55±0.04	0.61±0.06	0.60±0.05
Stearic, C18:0	16.88±0.19	17.22±0.32	16.38±0.36	17.00±0.28
Oleic, C18:1n-9	7.80±0.12	7.68±0.17	7.83±0.25	7.90±0.22
Vaccenic, C18:1n-7	2.36 [2.10, 2.73]	2.60 [2.17, 2.75]	2.24 [2.06, 2.89]	2.16 [1.99, 2.54]
Linoleic, C18:2n-6	23.89±0.42	23.66±0.75	24.69±0.74	23.31±0.64
DGLA, C20:3n-6	3.09±0.11	3.11±0.22	3.00±0.19	3.18±0.18
Arachidonic, C20:4n-6	10.89±0.27	11.25±0.49	11.06±0.35	10.26±0.51
EPA, C20:5n-3	0.30 [0.22, 0.42]	0.29 [0.22, 0.41]	0.33 [0.18, 0.43]	0.34 [0.23, 0.44]
Adrenic, C22:4n-6	0.41 [0.35, 0.51]	0.41 [0.35, 0.54]	0.46 [0.35, 0.55]	0.40 [0.31, 0.45]
Clupanodonic, C22:5n-3	0.56±0.02	0.55±0.02	0.53±0.03	0.59±0.05
DHA, C22:6n-3	2.79±0.11	2.79±0.18	2.51±0.17	3.07±0.23
D5D, AA/DGLA	3.59 [2.97, 4.83]	3.86 [2.82, 4.85]	3.59 [3.03, 5.21]	3.08 [2.84, 4.17]
D6D, DGLA/LA	0.13±0.01	0.14±0.01	0.13±0.01	0.14±0.01
AA/EPA	35.09 [26.18, 48.68]	36.74 [28.69, 53.13]	35.09 [26.87, 57.43]	29.90 [19.57, 40.34]
AA/DHA	4.08 [3.28, 4.98]	4.16 [3.32, 4.86]	4.42 [3.48, 6.35]	3.50 [2.52, 4.48]

Data are presented as Mean ± SEM or Median [25th, 75th percentile] depending on a distribution. Variance in distribution of the parameters was evaluated by the general linear model and Kruskal-Wallis analyses, for parametric and non-parametric approach, respectively; testing differences between parameter distributions across the study treatments with varying polyphenol content ($p>0.05$ for all parameters).
AJ, subjects drinking aronia juice with 1177.11 mg of polyphenols per 100 mL; MJ, subjects drinking aronia juice with 294.28 mg of polyphenols per 100 mL; PLB, subjects drinking polyphenol-lacking placebo beverage. AA, Arachidonic acid; DGLA, Dihomo-γ-linolenic acid; EPA, Eicosapentaenoic acid; DHA, Docosahexaenoic acid; D5D and D6D, predicted desaturase-5 and -6 enzymatic activities, respectively.

Table S3. Allele and genotype distribution of the *FADS2* genetic variants in subjects who are overweight.

	Chromosome Position	Reference allele homozygote *n* (%)	Heterozygote *n* (%)	Variant allele homozygote *n* (%)	Variant Allele Frequency Our Study/1000Genome	Chi-sq[a]	p-value[b]
**rs174593, T>C**	11:61851359	TT<br>27 (43.5)	TC<br>30 (48.4)	CC<br>5 (8.1)	0.32/0.21	1.14	0.286
**rs174616, G>A**	11:61861650	GG<br>14 (22.6)	GA<br>36 (58.1)	AA<br>12 (19.4)	0.48/0.48	2.63	0.105
**rs174576, C>A**	11:61836038	CC<br>23 (37.1)	CA<br>33 (53.2)	AA<br>6 (9.7)	0.36/0.36	2.26	0.133

[a]p value is associated with Chi-sq. [b]Chi-sq associated with test of compliance with Hardy-Weinberg equilibrium of balanced distribution of genotypes

Table S4. Distribution of 4-week changes (Δ) in body weight, BMI and long-chain polyunsaturated fatty acids in plasma phospholipids, respective of variant allele presence in the three *FADS2* variant places and regardless of treatment type.

	**rs174593**		**174616**		**174576**	
	Major homozygotes	Variant allele carriers	Major homozygotes	Variant allele carriers	Major homozygotes	Variant allele carriers
Δ BMI	-0.16±0.09	-0.12±0.07	-0.09±0.08	-0.15±0.07	-0.21±0.11	-0.10±0.06
Δ Body Weight	-0.40 [-1.20, 0.40]	-0.20 [-1.03, 0.43]	-0.40 [-0.95, 0.25]	-0.20 [-1.20, 0.50]	-0.20 [-1.30, 0.40]	-0.25 [-0.93, 0.43]
Δ Linoleic, C18:2 n-6	-0.85±0.58	-1.43±0.42	**0.47±0.89**	**-1.55±0.36**	**-0.33±0.54**	**-1.72±0.44**
Δ Arachidonic, C20:4 n-6	-1.12 [-2.19, 0.23]	-0.33 [-1.54, 0.60]	0.15 [-1.13, 0.78]	-1.10 [-1.82, 0.25]	-0.45 [-2.40, 0.54]	-1.10 [-1.65, 0.25]
Δ Adrenic, C22:4 n-6	-0.08 [-0.16, 0.03]	-0.07 [-0.17, 0.09]	0.00 [-0.17, 0.04]	-0.08 [-0.16, 0.04]	-0.07 [-0.13, 0.03]	-0.08 [-0.16, 0.09]
Δ EPA, C20:5 n-3	0.09 [-0.05, 0.29]	0.12 [-0.11, 0.36]	0.09 [0.02, 0.38]	0.10 [-0.10, 0.29]	0.09 [-0.10, 0.26]	0.10 [-0.07, 0.39]
Δ Clupanodonic C22:5 n-3	-0.07 [-0.16, 0.04]	0.02 [-0.14, 0.13]	-0.07 [-0.23, 0.06]	-0.05 [-0.14, 0.11]	-0.07 [-0.16, 0.06]	-0.05 [-0.14, 0.13]
Δ DHA, C22:6 n-3	0.04 [-0.45, 0.62]	0.12 [-0.32, 0.84]	0.22 [-0.60, 0.89]	0.10 [-0.29, 0.75]	-0.04 [-0.52, 0.44]	0.27 [-0.21, 0.87]
Δ D5D, AA/DGLA	0.14 [-0.13, 1.03]	-0.10 [-0.46, 0.45]	0.36 [-0.09, 1.02]	-0.01 [-0.47, 0.71]	**0.36 [-0.10, 1.13]**	**-0.10 [-0.46, 0.30]**
Δ D6D, DGLA/LA	-0.01 [-0.03, 0.01]	0.00 [-0.02, 0.02]	0.00 [-0.02, 0.01]	0.00 [-0.02, 0.02]	**-0.02 [-0.05, 0.01]**	**0.01 [-0.01, 0.02]**
Δ AA/EPA	-9.74 [-24.86, 2.17]	-5.16 [-26.97, 3.77]	-8.56 [-16.73, 0.26]	-8.13 [-27.02, 3.99]	-7.88 [-24.92, 6.94]	-8.56 [-26.79, 2.96]
Δ AA/DHA	-0.28 [-1.15, 0.25]	-0.26 [-0.95, 0.46]	-0.17 [-1.10, 0.75]	-0.28 [-0.99, 0.19]	0.05 [-0.77, 0.75]	-0.35 [-1.20, 0.08]

Data are presented as Mean ± SEM or Median [25th, 75th percentile] depending on a distribution. Variance in distribution of the parameters was evaluated by the general linear model and Kruskal-Wallis analyses, for parametric and non-parametric approach, respectively; testing differences between parameter distributions across *FADS2* genotype distributions. Bolded text denotes significantly different distribution of a parameter across genotypes. EPA, Eicosapentaenoic acid; DHA, Docosahexaenoic acid; D5D and D6D, predicted desaturase-5 and -6 enzymatic activities, respectively; DGLA, Dihomo-γ-linolenic acid; AA, Arachidonic acid.

Table S5. Associations between *FADS2* variants (rs174593, rs174616, rs174576) and changes in BMI and total body weight in subjects who are overweight (BMI≥25), across the study intervention groups with varying polyphenol intake.

		rs174593			rs174616			rs174576		
		ΔR²	B ± S.E.	*p*	ΔR²	B ± S.E.	*p*	ΔR²	B ± S.E.	*p*
					**Δ BMI**					
M1	**TS**	0.000	0.01±0.09	0.890	0.006	-0.05±0.08	0.541	0.016	0.09±0.0	0.329
M2		0.041	0.02±0.09	0.835	0.038	-0.04±0.09	0.638	0.053	0.12±0.0	0.193
M1	**AJ**	0.001	-0.03±0.20	0.871	0.021	-0.09±0.15	0.522	0.018	0.10±0.1	0.555
M2		0.182	0.07±0.20	0.738	0.159	0.03±0.16	0.840	0.265	0.27±0.1	0.122
M1	**MJ**	0.096	0.22±0.17	0.197	0.033	0.14±0.18	0.456	0.02	0.10±0.1	0.566
M2		0.155	0.25±0.17	0.155	0.141	0.14±0.18	0.434	0.185	0.20±0.1	0.283
M1	**PLB**	0.102	-0.18±0.13	0.181	0.074	-0.15±0.13	0.260	0.004	0.04±0.1	0.789
M2		0.04	-0.20±0.14	0.184	0.038	-0.17±0.15	0.263	0.035	0.05±0.1	0.749
					**Δ Body Weight**					
M1	**TS**	0.000	0.04±0.27	0.879	0.006	-0.16±0.26	0.545	0.019	0.29±0.2	0.288
M2		0.047	0.06±0.28	0.834	0.044	-0.12±0.26	0.645	0.061	0.39±0.2	0.159
M1	**AJ**	0.004	-0.19±0.66	0.774	0.019	-0.30±0.48	0.541	0.024	0.40±0.5	0.491
M2		0.191	0.16±0.65	0.805	0.176	0.13±0.51	0.797	0.292	0.99±0.5	0.088
M1	**MJ**	0.105	0.68±0.48	0.175	0.034	0.40±0.52	0.449	0.020	0.30±0.5	0.564
M2		0.141	0.73±0.48	0.152	0.134	0.41±0.51	0.431	0.180	0.59±0.5	0.275
M1	**PLB**	0.099	-0.53±0.39	0.190	0.071	-0.45±0.39	0.269	0.003	0.09±0.4	0.823
M2		0.041	-0.60±0.43	0.189	0.039	-0.52±0.45	0.266	0.033	0.12±0.4	0.793

M1, Crude regression model; M2, further adjusted for gender and habitual n-6/n-3 intake. TS, Total study sample; AJ, subjects drinking aronia juice with 1177.11 mg of polyphenols (gallic acid equivalents, GAE) per 100 mL; MJ, subjects drinking aronia juice with 294.28 mg of polyphenols (GAE) per 100 mL; PLB, subjects drinking polyphenol-lacking placebo beverage. p, probability values associated with unstandardized coefficients (B) upon multivariate-adjusted, hierarchical linear regression.
